# Anti-tumor effect of inhibition of IL-6 signaling in mucoepidermoid carcinoma

**DOI:** 10.18632/oncotarget.4477

**Published:** 2015-06-15

**Authors:** Daiki Mochizuki, April Adams, Kristy A. Warner, Zhaocheng Zhang, Alexander T. Pearson, Kiyoshi Misawa, Scott A. McLean, Gregory T. Wolf, Jacques E. Nör

**Affiliations:** ^1^ Department of Restorative Sciences, University of Michigan School of Dentistry, Ann Arbor, Michigan, USA; ^2^ Department of Otolaryngology/Head Neck Surgery, Hamamatsu University School of Medicine, Hamamatsu, Shizuoka, Japan; ^3^ Division of Hematology/Oncology, Department of Internal Medicine, University of Michigan School of Medicine, Ann Arbor, Michigan, USA; ^4^ Department of Otolaryngology, University of Michigan School of Medicine, Ann Arbor, Michigan, USA; ^5^ Department of Biomedical Engineering, University of Michigan College of Engineering, Ann Arbor, Michigan, USA; ^6^ Comprehensive Cancer Center, University of Michigan, Ann Arbor, Michigan, USA

**Keywords:** salivary gland cancer, tumorigenesis, tocilizumab, IL-6R, tumor initiating cells

## Abstract

Mucoepidermoid carcinoma (MEC) is the most frequent malignant salivary gland cancer. Response to chemoradiotherapy is modest, and therefore radical surgery remains the standard-of-care. Emerging evidence suggests that Interleukin (IL)-6 signaling correlates with the survival of cancer stem cells and resistance to therapy. Here, we investigated whether inhibition of IL-6 receptor (IL-6R) signaling with tocilizumab (humanized anti-human IL-6R antibody) sensitizes MEC to chemotherapy using human mucoepidermoid carcinoma cell lines (UM-HMC) and correspondent xenograft models. *In vitro*, we observed that tocilizumab inhibited STAT3 phosphorylation but had no measurable effect in MEC cell viability (UM-HMC-1,-3A,-3B). In contrast, the anti-tumor effect of single agent tocilizumab on MEC xenografts was comparable to paclitaxel or cisplatin. Combination of tocilizumab with cisplatin or paclitaxel enhanced the inhibitory effect of chemotherapy on xenograft growth (*P* < 0.05), time to failure (*P* < 0.01), decreased vascular endothelial growth factor (VEGF) expression and tumor microvessel density (*P* < 0.05) without added systemic toxicities. Notably, tocilizumab decreased the fraction of MEC cancer stem cells (ALDH^high^CD44^high^) *in vitro*, and prevented paclitaxel-induced increase in the fraction of cancer stem cells *in vivo* (*P* < 0.05). Collectively, these findings demonstrate that tocilizumab enhances the anti-tumor effect of conventional chemotherapy in preclinical models of mucoepidermoid carcinoma, and suggest that patients might benefit from combination therapy with an inhibitor of IL-6R signaling and chemotherapeutic agent such as paclitaxel.

## INTRODUCTION

Salivary gland cancers are complex and diverse malignancies comprised of 24 morphologically different neoplasms [1-4]. Mucoepidermoid carcinoma (MEC) is the most common malignant salivary gland cancer, histologically characterized as a glandular epithelial neoplasm containing mucous, intermediate, and epidermoid cells [4-6]. Histological grading correlates well with clinical behavior. As such, the 5-year survival for low or intermediate grade tumors is 85-98%, while for high grade tumors is only 22-55% [6-11]. Mucoepidermoid carcinomas are characterized by relentless growth and resistance to systemic therapy and radiotherapy. Therefore, the most effective therapy remains radical surgery, which is typically accompanied by severe facial disfigurement, loss of function, and major consequences to the quality of life of patients [5, 12-16]. The development of a mechanism-based therapeutic strategy that sensitizes these tumors to chemotherapy can potentially enhance the survival and quality of life of patients with mucoepidermoid carcinoma.

Interleukin-6 (IL-6) enhances the survival of cancer stem cells (CSC) in several tumors including glioma and breast cancer [17,18], and increases the conversion of non-cancer stem cells into cancer stem cells in breast cancer models [19]. Downstream mediators of IL-6 signaling (*e.g.* JAK/STAT3, PI3K, NF-κB) strongly correlate with the pathobiology of several malignancies [20-25]. In head and neck cancer, serum IL-6 levels correlate with poor prognosis [26, 27]. We have recently showed that cancer stem cells reside in the perivascular niche of head and neck squamous cell carcinomas [28], and that endothelial cell-secreted IL-6 enhances the survival, self-renewal, and tumorigenic potential of cancer stem cells [29]. We also observed that cisplatin treatment enhances the fraction of cancer stem cells in head and neck tumors [30]. We have recently observed that salivary mucoepidermoid carcinomas contain a sub-population of uniquely tumorigenic cancer stem cells, defined as ALDH^high^CD44^high^ cells. It is believed that cancer stem cells play a critical role in resistance to therapy in many glandular malignancies. However, it is unclear if IL-6 signaling is involved in the survival of cancer stem cells and the resistance to chemotherapy observed in patients with mucoepidermoid carcinoma.

Progress in the development of effective therapies for mucoepidermoid carcinoma has been hindered by the lack of experimental models. However, the recent characterization of mucoepidermoid carcinoma cell lines and accompanying xenograft models generated from patients with resistant disease [28] has finally enabled mechanistic studies and the testing of new therapies. Here, we evaluated the anti-tumor effect of tocilizumab, a humanized anti-human IL-6R antibody, in combination with conventional chemotherapy (cisplatin or paclitaxel) in preclinical models of mucoepidermoid carcinoma. We observed that therapeutic inhibition of IL-6R with tocilizumab enhanced the *in vivo* anti-tumor effect of both conventional chemotherapeutic agents tested here, despite having no direct effect on the survival of unsorted mucoepidermoid carcinoma cells *in vitro*. Notably, tocilizumab treatment caused a decrease in the microvessel density of xenograft tumors, and prevented paclitaxel-induced increase in the fraction of cancer stem cells. Collectively, these data demonstrate that therapeutic inhibition of IL-6R signaling with tocilizumab potentiates the anti-tumor effect of conventional chemotherapeutic drugs in preclinical models of mucoepidermoid carcinoma, and suggests that patients might benefit from the combined administration of tocilizumab and paclitaxel.

## RESULTS

### Expression of key mediators of IL-6 signaling in mucoepidermoid carcinoma

To begin to understand the therapeutic potential of targeting IL-6 signaling in mucoepidermoid carcinomas, we evaluated the expression of key components of the IL-6 pathway in primary human tumors and in our xenograft models (Supplementary Figure S1). We observed that the xenograft tumors generated with UM-HMC cell lines reproduced the typical morphology of primary human tumors, with areas of predominantly cystic, while other areas with more of a solid appearance (Supplementary Figure S1A). Immunofluorescence staining revealed that the IL-6 ligand, both receptors (*i.e.* IL-6R, gp130) and the key downstream effector pSTAT3 are highly expressed in these tumors (Supplementary Figure S1B and S1C). Notably, both human and xenograft tumors showed largely similar patterns of expression of these molecules (Supplementary Figure S1C). These descriptive results suggested that IL-6 could potentially play a significant role in the pathobiology of mucoepidermoid carcinoma, and encouraged us to perform developmental therapeutic studies with tocilizumab, a humanized anti-IL-6R antibody that has been approved by the FDA for treatment of rheumatoid arthritis since 2010.

### Tocilizumab inhibits the growth of mucoepidermoid carcinomas

In pilot experiments, we observed that single agent tocilizumab inhibited tumor growth to the same extent as single agent paclitaxel or cisplatin (Supplementary Figure S2A and S2C). While tocilizumab was well tolerated without causing a noticeable decrease in mouse weight, we observed a 10% weight loss in mice that received 20 mg/kg paclitaxel (Supplementary Figure S2A). Notably, the combination of tocilizumab with paclitaxel or cisplatin potentiated the overall effect of therapy leading to a tumoristatic effect without added toxicities (Supplementary Figure S2). The results of this pilot experiment suggested that IL-6R inhibition with tocilizumab have a therapeutic effect in preclinical models of mucoepidermoid carcinoma, and informed our decision to decrease the dose of paclitaxel to 15 mg/kg for the remaining studies.

**Figure 1:**
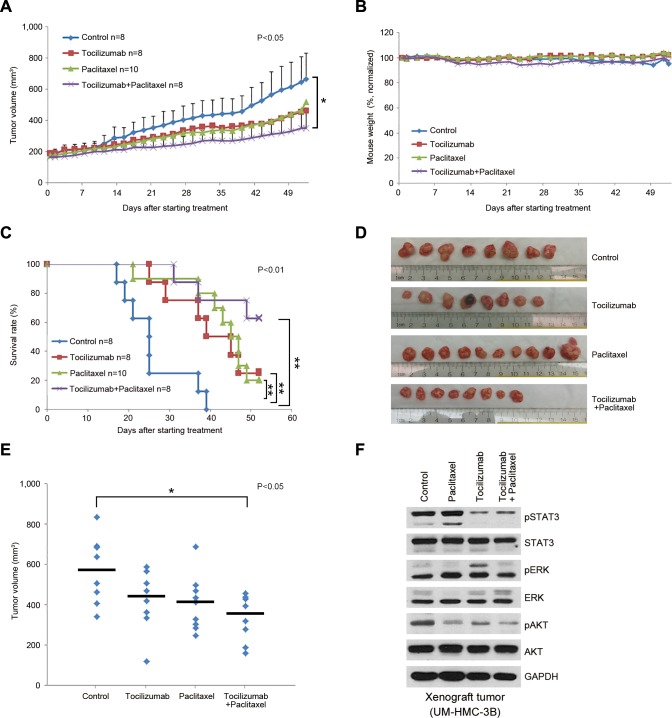
Effect of tocilizumab and/or paclitaxel in preclinical models of mucoepidermoid carcinoma Xenograft tumors were generated in immunodeficient mice upon transplantation of UM-HMC-3B cells. When tumors reached 200 mm^3^, mice were randomized into the 4 different treatment regimens as follows: 5 mg/kg IgG control, 5 mg/kg tocilizumab, 15 mg/kg paclitaxel, or 15 mg/kg paclitaxel combined with 5 mg/kg tocilizumab weekly via i.p. injection. **A.** Graph depicting tumor volume over time until the first tumors in the control group reached the cutoff size of 1,000 mm^3^. Tocilizumab with paclitaxel group has a significant effect on tumor volume compared with control group (*P* < 0.05). **B.** Graph depicting mouse weight during treatment. Data were normalized against pre-treatment weight. **C.** Kaplan-Meier analysis using as criterion for failure a 2-fold increase in tumor volume as compared with pre-treatment tumor volume. Paclitaxel with tocilizumab group extended time to failure significantly compared with control group, and single drug groups, tocilizumab and paclitaxel, also extended compared with control group (*P* < 0.01). **D.** Macroscopic view of xenografts tumors upon retrieval from the mice. **E.** Graph depicting the volume of each individual xenograft tumor. Asterisk (*) depicts a statistical difference between control and tocilizumab + paclitaxel group (*P* < 0.05). **F.** Western blot for phosphorylated and total STAT3, AKT, and ERK in the tumors treated with tocilizumab and/or paclitaxel as compared to vehicle control. Tumors were harvested 10 days after last dose of drugs.

**Figure 2:**
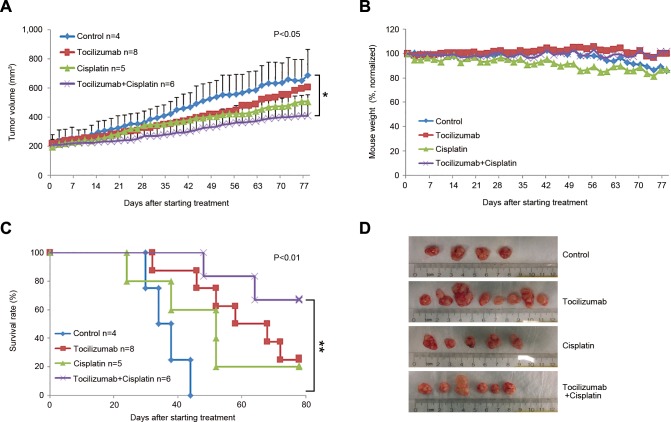
Effect of tocilizumab and/or cisplatin in preclinical models of mucoepidermoid carcinoma Xenograft tumors were generated in immunodeficient mice upon transplantation of UM-HMC-3B cells. When tumors reached 200 mm^3^, mice were randomized into the 4 different treatment regimens as follows: 5 mg/kg IgG control, 5 mg/kg tocilizumab, 15 mg/kg cisplatin, or 15 mg/kg cisplatin combined with 5 mg/kg tocilizumab weekly via i.p. injection. **A.** Graph depicting tumor volume over time until the first tumors in the control group reached the cutoff size of 1,000 mm^3^. Tocilizumab with cisplatin group has a significant effect on tumor volume compared with control group (*P* < 0.05). **B.** Graph depicting mouse weight during treatment. Data were normalized against pre-treatment weight. **C.** Kaplan-Meier analysis using as criterion for failure a 2-fold increase in tumor volume as compared with pre-treatment tumor volume. Cisplatin with tocilizumab group extended time to failure significantly compared with control group (*P* < 0.01). **D.** Macroscopic view of xenografts tumors upon retrieval from the mice.

When we repeated these experiments using a larger sample size (*n* = 8-10), the overall trends were similar to those observed in the pilot experiment (Figure 1). We observed that tocilizumab with paclitaxel or cisplatin group had a significant effect on tumor volume compared with control group (Figure 1A and 2A, *P* < 0.05), and single agent tocilizumab showed significant tumor growth inhibition, similar to single agent paclitaxel (Figure 1A and 1C-1E) or cisplatin (Figure 2A, 2C and 2D), without noticeable systemic toxicities (Figure 1B and 2B). Western blots of the tumor tissues retrieved from the mice at the end of the experiments revealed that tocilizumab, but not paclitaxel, inhibited the main downstream effector of IL-6 signaling, *i.e.* phosphorylated STAT3 (Figure 1F). Interestingly, tocilizumab and/or paclitaxel inhibited the AKT signaling pathway, a key regulator of tumor cell survival (Figure 1F). Kaplan-Meier analyses using as criterion for “event” a 2-fold increase in tumor volume as compared to pre-treatment size, showed significant tumor inhibition for single agent Tocilizumab (*P* < 0.01), when compared to vehicle control (Figure 1C). Notably, we observed even more marked tumor inhibition when tocilizumab was used in combination with paclitaxel (Figure 1C) or cisplatin (Figure 2C), when compared to controls (*P* < 0.01). Calculation of tumor growth inhibition (TGI) index confirmed previous results, demonstrating an overall anti-tumor effect for Tocilizumab that was comparable with single agent conventional chemotherapy, and a significant potentiation of this effect when the targeted drug was combined with Paclitaxel (Supplementary Figure S3).

### Tocilizumab is not cytotoxic to mucoepidermoid carcinoma cells *in vitro*

**Figure 3:**
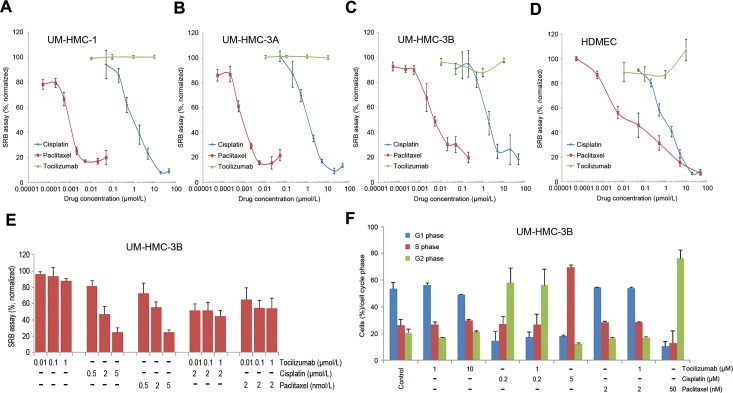
Tocilizumab is not cytotoxic to mucoepidermoid carcinoma cells *in vitro* **A.**-**D.** Graphs depicting the cytotoxicity of cisplatin, paclitaxel, and tocilizumab in UM-HMC-1, UM-HMC-3A, and UM-HMC-3B, and HDMEC, as determined by SRB assays. Cells were exposed to cisplatin, paclitaxel or tocilizumab for 72 hours. Data were normalized against vehicle control and initial plating density. **E.** Graph depicting the effect of tocilizumab combination with cisplatin or paclitaxel in UM-HMC-3B cells. Each panel is representative of at least three independent experiments, done in quadruplicate wells per condition. **F.** Graph depicting the effect of the drugs on cell cycle. Cells were exposed to cisplatin, paclitaxel and/or tocilizumab for 24 hours. Cells were stained with propidium iodide and subjected to flow cytometry for cell cycle analysis.

To understand potential mechanisms underlying the *in vivo* anti-tumor effect of tocilizumab, we performed a series of *in vitro* experiments. Cell viability assays using a panel of mucoepidermoid carcinoma cell lines (UM-HMC-1,-3A,-3B) and primary human endothelial cells (HDMEC) revealed that the overall IC_50_ for cisplatin ranged between 0.99-1.75 μmol/L, while the IC_50_ for paclitaxel was 24 nmol/L in endothelial cells and 0.75 to 2.89 nmol/L in UM-HMC cell lines (Figure 3A-3D). In contrast, Tocilizumab had no measurable cytotoxic effect in the panel of cells evaluated here (Figure 3A-3D). We also investigated the effect of combination therapy with cisplatin or paclitaxel and observed that tocilizumab does not have a significant impact in cell density (Figure 3E) or cell cycle (Figure 3F). We repeated these experiments with the other 2 UM-HMC cell lines and with endothelial cells, and observed similar results (data not shown). Collectively, these experiments indicated that tocilizumab does not have a direct effect on the survival of human mucoepidermoid carcinoma cells.

### Tocilizumab decreases tumor microvessel density

**Figure 4:**
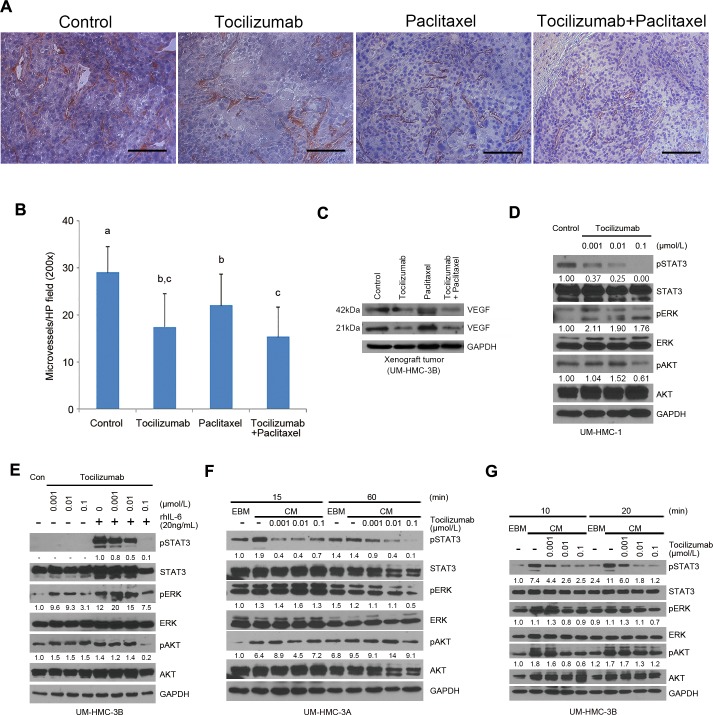
Tocilizumab inhibits tumor microvessel density and STAT3 activity **A.** Representative photomicrographs of histologic sections stained for Factor VIII (*red*) and counterstained with Hematoxylin to identify blood vessels in xenograft tumors. Scale bar represents 100 μm at x200 magnification. **B.** Graph depicting microvessel density in 5 random fields per tumor from 5 tumors per group. Different low-case letters indicate significant differences at *P* < 0.01. **C.** Western blot for VEGF in xenograft tumors. **D.**-**G.** Western blots for phosphorylated and total STAT3, AKT, and ERK in UM-HMC cells. UM-HMC-1 cells were incubated with tocilizumab at a range of concentrations from 0.1 to 0.001 μmol/L for 24 hours **D.** UM-HMC-3B cells were preincubated with tocilizumab at a same range of concentration of 0.1 to 0.001 μmol/L for 1 hour, and then incubated with or without 20 ng/mL rhIL-6 for 30 min **E.** Cells (UM-HMC-3A, UM-HMC-3B) were preincubated with tocilizumab at a range of concentrations of 0.1 to 0.001 μmol/L for 2 hours, and then changed medium to endothelial cell conditioned medium (CM) or control unconditioned medium (EBM) in presence of increasing concentrations of tocilizumab **F.** and **G.** Quantification of band density was performed with the NIH ImageJ software, using total STAT3, ERK and AKT as reference for band density, and using untreated controls to normalize the data.

As we were analyzing histologically the xenograft tumors, we observed that perhaps therapeutic inhibition of IL-6R signaling with tocilizumab had an anti-angiogenic effect *in vivo* despite the lack of a direct cytotoxic effect to endothelial cells *in vitro*. Indeed, we observed a decrease (*P* < 0.01) in tumor microvessel density when tocilizumab was used alone or in combination with paclitaxel (Figure 4A and 4B). Furthermore, VEGF expression was decreased when single agent tocilizumab or combination of tocilizumab with paclitaxel group was used *in vivo* (Figure 4C), providing a putative mechanism for the anti-angiogenic effect of tocilizumab described above. We have shown that endothelial cells secrete IL-6, CXCL8, and EGF that induce phosphorylation of STAT3, AKT, and ERK in tumor cells, and that these phosphorylation events enhance tumor cell survival and migration [31-33]. We have recently demonstrated that endothelial cell-secreted IL-6 regulates the rate of tumor growth via STAT3 signaling [34]. Considering the inhibitory effect of tocilizumab on constitutively active STAT3 in UM-HMC-1 cells (Figure 4D) and on IL-6-induced pSTAT3 in UM-HMC-3B (Figure 4E), we performed a series of experiments using endothelial cell conditioned medium as a source of IL-6 in an attempt to mimic endothelial cell-cancer cell interactions observed in the perivascular area [31-34]. We observed a potent induction of STAT3 phosphorylation upon exposure of mucoepidermoid carcinoma cells (UM-HMC-3A,-3B) to endothelial-conditioned medium, which was partially blocked in a dose-dependent manner with increasing concentrations of tocilizumab (Figure 4F and 4G). We concluded from these experiments that tocilizumab interferes with the crosstalk between endothelial cells and mucoepidermoid carcinoma cells, with a net anti-angiogenic effect that can explain, at least in part, the overall anti-tumor effect of this drug.

### Effect of Tocilizumab on cancer stem cells

**Figure 5:**
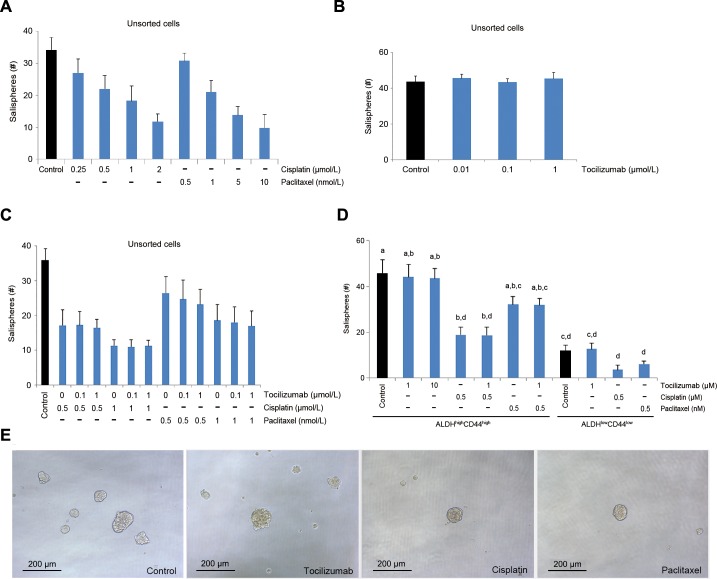
Effect of tocilizumab on cancer stem cells *in vitro* Salisphere assays were performed with UM-HMC-3B cells seeded in 96 well plates. **A.** and **B.** Graph depicting the number of salispheres when non-sorted (bulk) cells were exposed to cisplatin, paclitaxel **A.** or tocilizumab **B.** for 5 days. **C.** Graph depicting the number of salispheres formed by non-sorted (bulk) cells exposed to cisplatin or paclitaxel in combination with tocilizumab for 5 days. **D.** Graph depicting the number of salispheres formed by FACS-sorted ALDH^high^CD44^high^ or ALDH^low^CD44^low^ cells exposed to cisplatin or paclitaxel in combination with tocilizumab for 5 days. Different low-case letters indicate significant difference among groups (*P* < 0.05). **E.** Photomicrographs of representative salispheres using bulk cells. Scale bars represent 200 μm at x100 magnification.

We have recently demonstrated the function of a small sub-population of cells endowed with self-renewal and uniquely tumorigenic capacity, namely cancer stem cells, in mucoepidermoid carcinomas. Testing of several putative marker combinations demonstrated that cancer stem cells are identified in mucoepidermoid carcinomas by high aldehyde dehydrogenase (ALDH) activity and CD44 expression (ALDH^high^CD44^high^). To understand the effect of tocilizumab in mucoepidermoid carcinoma stem cells *in vitro*, we performed sphere assays as we described [35]. While paclitaxel and cisplatin mediated dose-dependent decreases in the number of salispheres generated with unsorted UM-HMC-3B cells (Figure 5A), tocilizumab did not show a measurable effect (Figure 5B). Drug combinations with tocilizumab and paclitaxel or cisplatin followed similar trends of single drug experiments without any noticeable effect for tocilizumab in both, unsorted cells (Figure 5C) or UM-HMC-3B cells FACS-sorted for ALDH and CD44 (Figure 5D). As expected, the number of spheres formed by untreated ALDH^high^CD44^high^ cells was higher than by ALDH^low^CD44^low^ cells (Figure 5D). Collectively, these studies revealed an inhibitory effect for paclitaxel and for cisplatin, and lack of measurable effect for tocilizumab, in cancer stem cells using the *in vitro* sphere assay in ultra-low attachment plates (Figure 5E).

**Figure 6:**
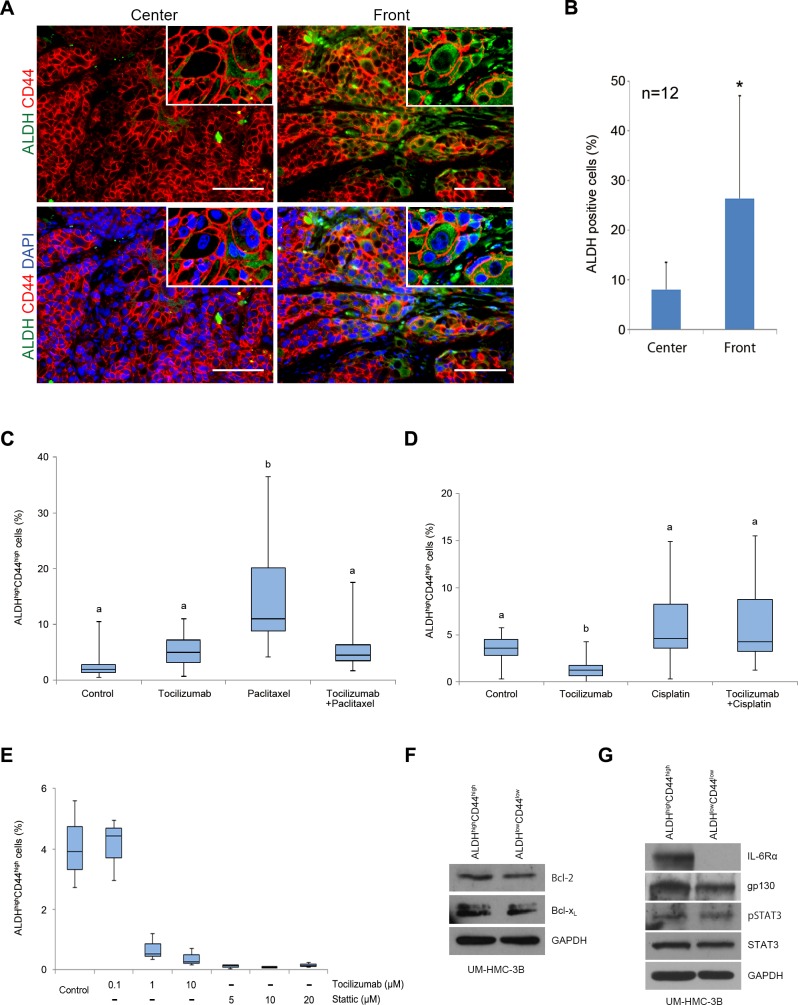
Effect of tocilizumab and paclitaxel on cancer stem cells *in vivo* **A.** Representative images obtained by immunofluorescence for ALDH1 (green) and CD44 (red) in the center of human tumors and in the invasive front. Upper row pictures depict images without DAPI (blue), and lower pictures are with DAPI (x200). Scale bars represent 100 μm. **B.** Graph depicting the percentage of ALDH positive cells in the center and in the invasive front of the tumors. ALDH-positive cells were counted in 3 random fields from 4 tumors per condition. Asterisk (*) depicts a statistical difference in the percentage of ALDH-positive cells when center and invasive fronts are compared (*P* < 0.05). **C.** and **D.** Graphs depicting the fraction of cancer stem cells (ALDH^high^CD44^high^) identified by flow cytometry in tumors treated with tocilizumab and/or paclitaxel **C.** or tocilizumab and/or cisplatin **D.** Different low-case letters indicate significant differences among groups (*P* < 0.05). **E.** Graph depicting the fraction of cancer stem cells (ALDH^high^CD44^high^) identified by flow cytometry in UM-HMC-3B cells treated with tocilizumab or Static for 24 hours. **F.** and **G.** Western blots for Bcl-2 and Bcl-x_L_ **F.** or phosphorylated and total STAT3, IL-6R, and gp130 **G.** in cancer stem cells (ALDH^high^CD44^high^) or control cells (ALDH^low^CD44^low^) sorted from UM-HMC-3B cell line.

To investigate the effect of tocilizumab on putative cancer stem cells *in vivo*, we performed immunofluorescence staining for ALDH1 and CD44 in mucoepidermoid carcinoma xenografts (Figure 6A). As expected, ALDH1 staining was localized in the cytoplasm and was limited to a small sub-set of cells, while CD44 was localized in the cell membrane and presented a more generalized staining pattern (Figure 6A). Interestingly, the fraction of ALDH-positive cells was significantly higher (*P* < 0.05) in the tumor invasive fronts, as compared to the central areas (Figure 6A and 6B). To quantify the effect of therapy on the fraction of cancer stem cells *in vivo*, we digested the xenograft tumors, generated single-cell suspensions, stained them for ALDH activity and CD44 expression, and sorted ALDH^high^CD44^high^ cells by flow cytometry. Therapy with tocilizumab by itself showed mixed results. In one experiment it did not have a significant effect on the fraction of ALDH^high^CD44^high^ cells (Figure 6C), while in the other experiment it decreased the fraction of cancer stem cells (Figure 6D). Paclitaxel treatment mediated a significant increase (*P* < 0.01) in the fraction of ALDH^high^CD44^high^ cells (Figure 6C). However, adding tocilizumab to the treatment with paclitaxel mediated a return of the fraction of ALDH^high^CD44^high^ cells to baseline levels (Figure 6C). Cisplatin treatment did not cause a significant increase in the fraction of ALDH^high^CD44^high^ cells, and its combination with tocilizumab had no measurable consequences (Figure 6D). We conclude from this set of experiments that the majority of the tumorigenic cancer stem cells are in the invasive fronts of xenograft mucoepidermoid carcinomas, and that tocilizumab inhibits paclitaxel-induced enrichment of the fraction of these cancer stem cells.

To further understand the effect of tocilizumab in the cancer stem cell fraction, we performed *in vitro* experiments with UM-HMC-3B cells treated with tocilizumab or with Stattic, a small molecule inhibitor of STAT3. Tocilizumab treatment resulted in a dose-dependent reduction in the fraction of cancer stem cells (Figure 6E). Interestingly, direct inhibition of STAT3 with Stattic also decreased the fraction of cancer stem cells (Figure 6E). These effects were not simply due to differential expression of pro-survival proteins of the Bcl-2 family in cancer stem cells, as compared to control cells (Figure 6F). Rather, the tocilizumab-mediated decrease in the fraction of cancer stem cells might be attributable to the differential expression of the receptor IL-6R, which is strongly expressed in ALDH^high^CD44^high^ cells when compared to ALDH^low^CD44^low^ cells (Figure 6G). Interestingly, the expression of gp130, total and phospho-STAT3 was similar in both cancer stem cells and non-stem cells. Collectively, these results demonstrate that the effect of tocilizumab in the cancer stem cell fraction correlates with the increased expression of its target receptor (IL-6R) in mucoepidermoid carcinoma stem cells.

## DISCUSSION

IL-6 plays a major role in the pathobiology of cancer. It is a key regulator of tumor cell proliferation, invasion and survival; it regulates the crosstalk between tumor cells and inflammatory cells, as well as vascular endothelial cells; and it enhances the survival and plasticity of cancer stem cells [17-25]. Such findings provided the rationale for clinical trials using targeted inhibitors of IL-6 in patients with cancer (*e.g.* ovarian cancer, multiple myeloma, renal cell carcinoma, prostate cancer) that showed promising results [24, 36-40]. We have recently demonstrated that endothelial cell-secreted IL-6 regulates tumor growth [34] and the tumorigenic potential of cancer stem cells in head and neck squamous cell carcinomas [29]. We have also observed that mucoepidermoid carcinomas contain a population of uniquely tumorigenic cancer stem cells. However, we do not know the effect of inhibition of IL-6 signaling in mucoepidermoid carcinomas. Here, we showed that therapeutic inhibition of IL-6R with tocilizumab has no measurable effect on mucoepidermoid carcinoma cell viability, cell cycle, or sphere formation *in vitro*. Surprisingly, the *in vivo* studies showed very different results. Tocilizumab has a significant anti-tumor effect in xenograft mucoepidermoid carcinomas that correlated with potent inhibition of STAT3 signaling and inhibition of tumor angiogenesis. Single agent tocilizumab effect was similar to single agent paclitaxel or cisplatin, but tocilizumab was very much better tolerated by the mice. Tocilizumab further potentiated the anti-tumor effect of paclitaxel, and prevented paclitaxel-induced enrichment of cancer stem cells, without added toxicities. These results were correlated with a decrease in the cancer stem cell (ALDH^high^CD44^high^) fraction mediated by Tocilizumab *in vitro,* and with the observation the cancer stem cells express higher levels of IL-6R when compared to control cells. Collectively, these data unveiled IL-6 as a therapeutic target for salivary gland mucoepidermoid carcinomas.

Patients with mucoepidermoid carcinoma are treated with surgery, radiation, and chemotherapy with taxanes or platinum-based drugs [5, 12-14, 41]. Treatment with these drugs is typically accompanied by severe systemic toxicities. Here, we observed that tocilizumab alone had similar anti-tumor effects when compared with paclitaxel or cisplatin. Notably, tocilizumab has been FDA-approved since 2010 for rheumatoid arthritis with an excellent record of safety for patients. The results of this preclinical study support the concept that tocilizumab could serve as a low-toxicity alternative to conventional chemotherapy in selected patients with mucoepidermoid carcinoma. We went a step forward, and performed drug combination studies that revealed that tocilizumab enhanced the anti-tumor effect of conventional chemotherapy, particularly paclitaxel. Indeed, we observed a significant improvement in time to failure when tocilizumab was used together with paclitaxel, when compared to monotherapy or vehicle controls. Notably, paclitaxel by itself enriched the fraction of the highly tumorigenic cancer stem cells, as it has been also demonstrated for other chemotherapeutic agents in head and neck cancer [30]. In contrast, addition of tocilizumab to the treatment with paclitaxel brought the fraction of cancer stem cells down to baseline levels. These results further suggest that the proposed combined therapy might be beneficial to patients. Nevertheless, additional studies are necessary to understand the long-term benefits of cancer stem cell ablation in preclinical models of mucoepidermoid carcinoma.

We observed in several studies that tocilizumab is effective *in vivo* while has essentially no effect whatsoever *in vitro*. We speculate that this is due to its effect on angiogenesis and inflammation [42, 43], as well as its putative effect disrupting interactions between endothelial cells and cancer stem cells within the perivascular niches. There are several possible explanations for these intriguing results: A) We showed that endothelial cell-derived IL-6 is critical for tumor growth [30]. Considering the well-known anti-angiogenic effect of IL-6 inhibition [20], and our own results demonstrating that tocilizumab inhibited tumor angiogenesis, it is possible that the anti-tumor effect of tocilizumab is mediated, at least in part, by decreased supply of oxygen and nutrients to tumor cells. B) By disrupting perivascular niches, *i.e.* the site where cancer stem cells typically reside in carcinomas [28], tocilizumab may disrupt this crosstalk between endothelial cells and cancer stem cells. We have recently showed that endothelial cell-secreted IL-6 enhances the tumorigenic potential of cancer stem cells [29]. Therefore, part of the effect observed here with tocilizumab might be mediated by interfering in this important crosstalk within the perivascular niche *in vivo*. And C) Tocilizumab is a potent inhibitor of STAT3 phosphorylation *in vivo* (Figure 1F). Considering the critical role of STAT3 signaling in cancer (*e.g.* regulation of VEGF expression and tumor angiogenesis, regulation of pro-inflammatory cytokines), it is possible that the effect of tocilizumab *in vivo* is mediated indirectly via blockade of downstream events induced by STAT3 signaling [44-47]. Such processes are not observable *in vitro*, and suggest that a true appreciation of the mechanisms underlying the therapeutic potential of tocilizumab require *in vivo* studies, at least in models of mucoepidermoid carcinoma.

IL-6 is clearly not the only endothelial cell-derived factor that could potentially affect the behavior of mucoepidermoid carcinoma cells. Indeed, we have shown that endothelial cell-derived CXCL8 and EGF have significant effects on the survival and motility of head and neck squamous cell carcinoma cells [32, 33]. Here, we used endothelial cell conditioned medium as a physiological strategy to induce activation of major signaling pathways (*i.e.* STAT3, Akt, ERK) in mucoepidermoid carcinoma cells, to enable the study of potential effects of tocilizumab in these pathways. Surprisingly, despite the numerous cytokines/chemokines present in the endothelial cell conditioned medium, blockade of IL-6R signalling with Tocilizumab was sufficient to inhibit STAT3 phosphorylation below baseline levels (Figure 4F and 4G). Considering the prominent role of STAT3 signalling in the biology of cancer cells, these results provide further support to the therapeutic potential of IL-6R inhibition with tocilizumab in mucoepidermoid carcinoma.

In summary, patients with mucoepidermoid carcinoma have very few therapeutic options today, and most of them include radical surgical resection because these tumors simply do not respond to conventional chemotherapy [5, 12-16]. Here, we used recently characterized models of mucoepidermoid carcinoma and observed that single agent tocilizumab has similar anti-tumor effects as conventional chemotherapy, without significant systemic toxicities. Further, we observed that combination of tocilizumab with paclitaxel enhanced the anti-tumor effect without added toxicities, and prevented paclitaxel-induced accumulation of cancer stem cells. These results demonstrate the anti-tumor effect of tocilizumab, and suggest that patients with mucoepidermoid carcinoma might benefit from the targeted inhibition of IL-6R signaling.

## MATERIALS AND METHODS

### Cells and reagents

We used the University of Michigan-Human Mucoepidermoid Carcinoma cells (UM-HMC-1,-3A,-3B), a panel of cell lines recently generated and characterized by our laboratory [48]. Cells were cultured in Dulbecco's Modified Eagle's Medium (DMEM; Invitrogen, Carlsbad, CA, USA) supplemented with 10% Fetal Bovine Serum (FBS; Invitrogen), 1% 200 mM L-glutamine (Invitrogen), 20 ng/ml epidermal growth factor (EGF; Sigma-Aldrich, St. Louis, MO, USA), 400 ng/mL hydrocortisone (Sigma-Aldrich), 5 μg/ml insulin (Sigma-Aldrich), and 1% penicillin/streptomycin (Invitrogen) at 37°C with 5% CO_2_. Primary human dermal microvascular endothelial cells (HDMEC; Lonza, Walkersville, MD, USA) were cultured in endothelial cell growth medium-2 for microvascular cells (EGM2-MV; Lonza). The experimental drugs used here were a humanized anti-IL-6R antibody (Tocilizumab; Genentech, San Francisco, CA, USA), control IgG (Jackson Laboratories, West Grove, PA, USA), cisplatin (Acros organics, Fairlawn, NJ, USA), and paclitaxel (Sagent pharmaceuticals, Schaumburg, IL, USA).

### Sulforhodamine B (SRB) assay

SRB assays were performed to evaluate the effect of compounds on mucoepidermoid carcinoma cell viability, as described [49]. MEC cells (UM-HMC-1,-3A,-3B) were seeded at a density of 2×10^3^ cells per well in 96-well plates, allowed to adhere overnight, and treated with drugs (or controls) for 72 hours. Plates were read in a microplate reader at 560 nm (GENios; Tecan, Granz, Austria). Data were obtained from triplicate wells per condition and are representative of at least 3 independent experiments.

### Western blot analysis

UM-HMC cell lines were plated, serum starved overnight, treated with tocilizumab or Stattic (STAT3 inhibitor V, Calbiochem, San Diego, CA, USA) at the indicated concentrations, and exposed to 20 ng/ml rhIL-6 (R&D Systems, Minneapolis, MN, USA). NP-40 lysis buffer was used to prepare whole cell lysates that were resolved using PAGE. Membranes were incubated with the following primary antibodies for 1 hour at room temperature or overnight at 4°C: mouse anti-human phospho-STAT3, rabbit anti-human STAT3, rabbit anti-human phospho-ERK1/2, rabbit anti-human ERK1/2, rabbit anti-human phospho-AKT, rabbit anti-human AKT, rabbit anti-human Bcl-x_L_, rabbit anti-human gp130 (Cell Signaling, Beverly, MA, USA), rabbit anti-human IL-6Rα, rabbit anti-human VEGF (Santa Cruz Biotechnology, Santa Cruz, CA, USA), hamster anti-human Bcl-2 (BD Pharmingen, Franklin Lakes, NJ, USA); or mouse anti-GAPDH (Chemicon, Billerica, MA, USA).

### Flow cytometry

Cancer stem cells were identified as ALDH^high^CD44^high^ cells, as we described [28-30]. Single-cell suspensions were obtained from the digestion of xenograft tumors or from the UM-HMC-3B cell line. Cells were counted, resuspended at 2×10^6^ cells/ml PBS, and incubated with activated Aldefluor^®^ substrate (BAA) or the ALDH inhibitor (DEAB) for 45 minutes at 37°C, using the Aldefluor^®^ kit (StemCell; Vancouver, BC, Canada). Cells were exposed to anti-CD44 antibody (APC-Cat #559942, PE-Cat #550989) for 30 minutes at 4°C. Anti-HLA-ABC (PE-Cat #560168; BD Pharmingen) was used to separate human cells from mouse cells, and 7-AAD (Cat #00-6993-50; eBiosciences, San Diego, CA, USA) staining was used to exclude dying cells. To measure the effect of drugs on cell cycle, UM-HMC-3B cells were treated with tocilizumab, cisplatin, paclitaxel, or controls diluted in cultured medium. After 24 hours, cells were retrieved, exposed to a hypotonic solution of propidium iodide containing 0.1% sodium citrate, 25 μg/ml propidium iodide, 100 μg/ml RNase A, and 0.1% Triton X-100. Cell cycle analysis was performed by flow cytometry, as described [50].

### Salisphere assay

Non-adherent spheroids of mucoepidermoid carcinoma cells (named salispheres) were generated in ultra-low attachment 96-well plates (Corning; Corning, NY, USA) for 5 days with DMEM-F12 (Invitrogen) supplemented with 1% N2 supplement (Invitrogen), 1% GlutaMAX (Invitrogen), 1 μM dexamethasone (Sigma-Aldrich), 20 ng/ml EGF (Sigma-Aldrich), 20 ng/mL basic fibroblast growth factor (bFGF, Sigma-Aldrich), 10 μg/ml insulin (Sigma-Aldrich), and 1% penicillin/streptomycin (Invitrogen).

### SCID mouse model of human tumor angiogenesis

Porous poly-L-lactic acid scaffolds (6×6×1 mm) with an average pore diameter of 180 μm were fabricated, as we described [51]. Just before transplantation, scaffolds were seeded with 7×10^5^ HDMEC and 3×10^5^ UM-HMC-3B cells in a 1:1 Matrigel/EGM2-MV mix. Female severe combined immunodeficient mice (CB.17.SCID; Charles River Laboratory, Wilmington, MA, USA) were anesthetized with ketamine/xylazine, and two bilateral scaffolds were transplanted in the subcutaneous space of the dorsal region of each mouse. When the average tumor volume reached 200 mm^3^, mice were randomized into the following treatment conditions: 5 mg/kg IgG control, 5 mg/kg tocilizumab, 5 mg/kg cisplatin and/or 15 mg/kg paclitaxel administered weekly via intraperitoneal injection. When 2 drugs were combined (*e.g.* tocilizumab + paclitaxel), they were administered with a 12-hour interval between them. Tumors were surgically retrieved when they reached 1,000 mm^3^, fixed overnight in 10% buffered formalin (Fisher Scientific, Pittsburgh, PA, USA) at 4°C, and processed for immunohistochemistry. Tumor size was calculated using the formula: volume (mm^3^) = *L* × *W*^2^/2 (*L*, length; *W*, width). The care and treatment of experimental animals was in accordance with University of Michigan institutional guidelines.

### Immunofluorescence and Immunohistochemistry

Tissue sections were incubated at 60°C for 20 minutes, deparafinized in xylene twice for 10 minutes, washed with 100% ethanol twice for 5 minutes, and then rehydrated with graded ethanol. Antigen retrieval was performed with Target Retrieval Solution (Dako, Carpinteria, CA, USA) following manufacturer's instructions, and the slides were washed with PBS thrice at room temperature. Endogenous peroxidase activity was quenched by 10-minute incubation in 3% hydrogen peroxide in PBS. Primary antibodies against ALDH1 (Abcam, Cambridge, England), IL-6 (Abcam), IL-6R (Santa Cruz Biotechnology), gp130 (Santa Cruz Biotechnology), CD44 (Thermo Scientific, Rockford, IL, USA), and pSTAT3 (Cell Signaling) were prelabeled with Alexafluor 488 or 594 using a Zenon labeling kit, and Prolong Gold Anti-fade Reagent with DAPI (Invitrogen) was used to mount the slides. Immunofluorescence images were taken with a Nikon Eclipse 80i fluorescence microscope, and image processing was done with Q-Capture Pro 7 (Q-imaging) and NIH ImageJ. Alternatively, tissue sections were incubated in Target Retrieval Solution (Dako) for 20 min at 90-95°C, followed by incubation with primary antibody at 4°C overnight. Polyclonal anti-human factor VIII antibody (Thermo Scientific) was used to identify microvascular networks, as described [52]. Blood vessels were counted in 5 random fields/tumor from 5 tumors per experimental condition.

### Statistical analyses

Statistical analysis was performed using Welch's *t*-test, Kruskal-Wallis tests, or one-way ANOVA followed by appropriate post-hoc tests. Kaplan-Meier curves were analyzed with the Gehan's generalized Wilcoxon tests. Tumor regression was analyzed using a linear mixed model regression to analyze the repeated measurements on each tumor. Model fixed effects included time, tocilizumab, chemotherapy, and the interaction of treatments and time. Model random effects included both mouse and tumor position. For all models a continuous autoregressive correlation structure was used, which assumes more correlated variances among temporally proximate observations. A log-transformation of the outcome variable (tumor volume) was used because the tumor volumes follow an exponential increase. Size at study initiation was also controlled for. Analysis was performed using the “nlme” package in the statistical software program R version 3.1.0. (2008; R Development Core Team). R: A language and environment for statistical computing. R Foundation for Statistical Computing, Vienna, Austria. (http://www.R-project.org.) Statistical significance was determined at *P* < 0.05.

## SUPPLEMENTARY MATERIAL FIGURES


