# Supplementary material for: Anti-tumor effect of inhibition of IL-6 signaling in mucoepidermoid carcinoma
Source: Oncotarget. 2015 Jun 15;6(26):22822–35. doi: 10.18632/oncotarget.4477 (PMC4673202; doi:10.18632/oncotarget.4477)
Supplement: Supplementary file 1 [file oncotarget-06-22822-s001.pdf]

Anti-tumor effect of inhibition of IL-6 signaling in mucoepidermoid carcinoma

Supplementary Material

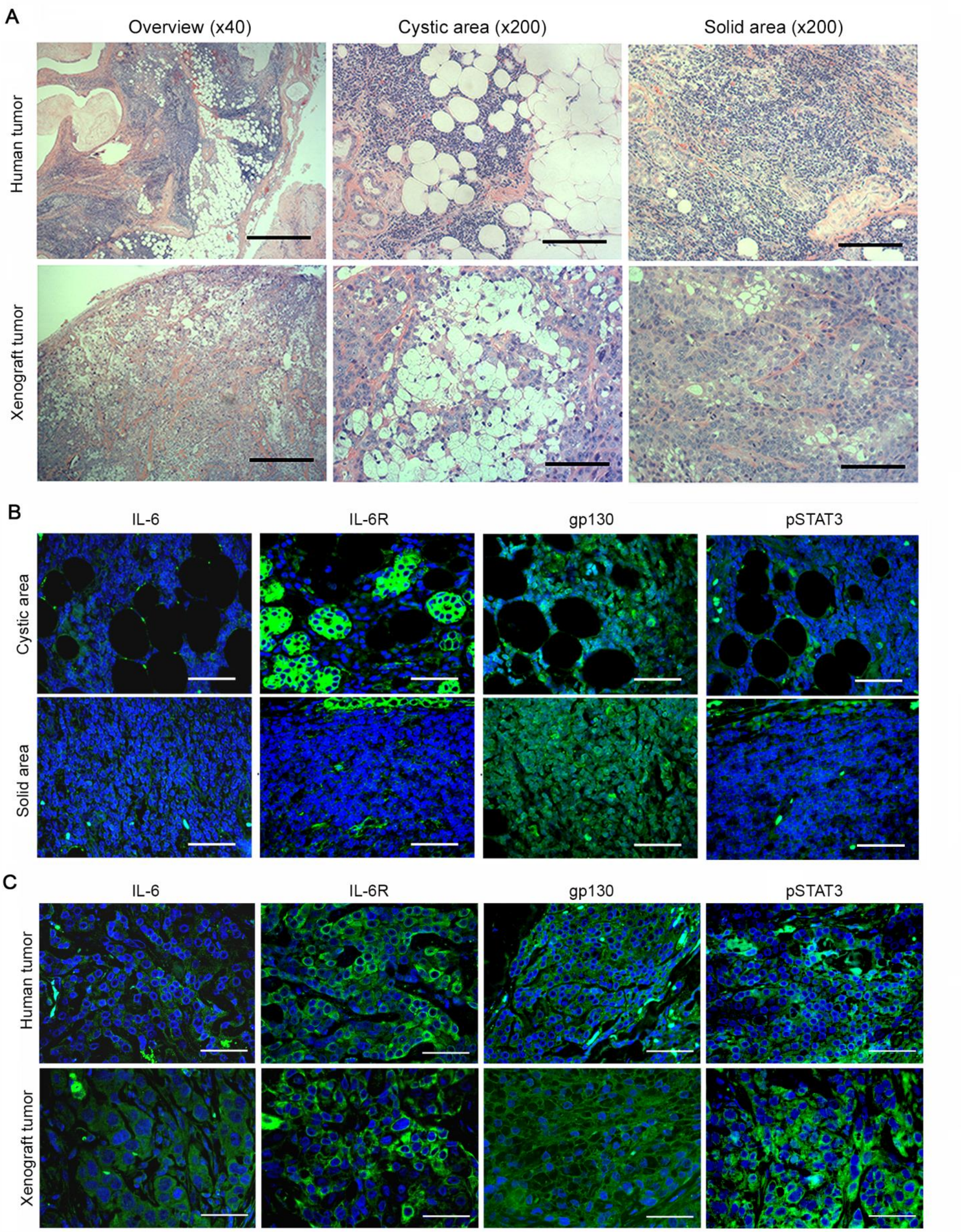

Supplementary Figure S1: Expression of key mediators of IL-6 signaling in mucoepidermoid carcinoma. A, representative images of histologic sections of human and xenograft mucoepidermoid carcinomas stained with hematoxilin and eosin. Photomicrographs were taken in areas characterized as predominantly cystic or solid. B, expression of IL-6, IL-6R, gp130, and phosphorylated STAT3 as determined by immunofluorescence in cystic and solid areas of human tumors (x400). C, expression levels of IL-6, IL-6R, gp130, pSTAT3 as determined by immunofluorescence in human and xenograft tumors (x400). Target antibodies are shown in green, while cell nuclei were stained by DAPI (blue). Scale bar represents 500 µm.

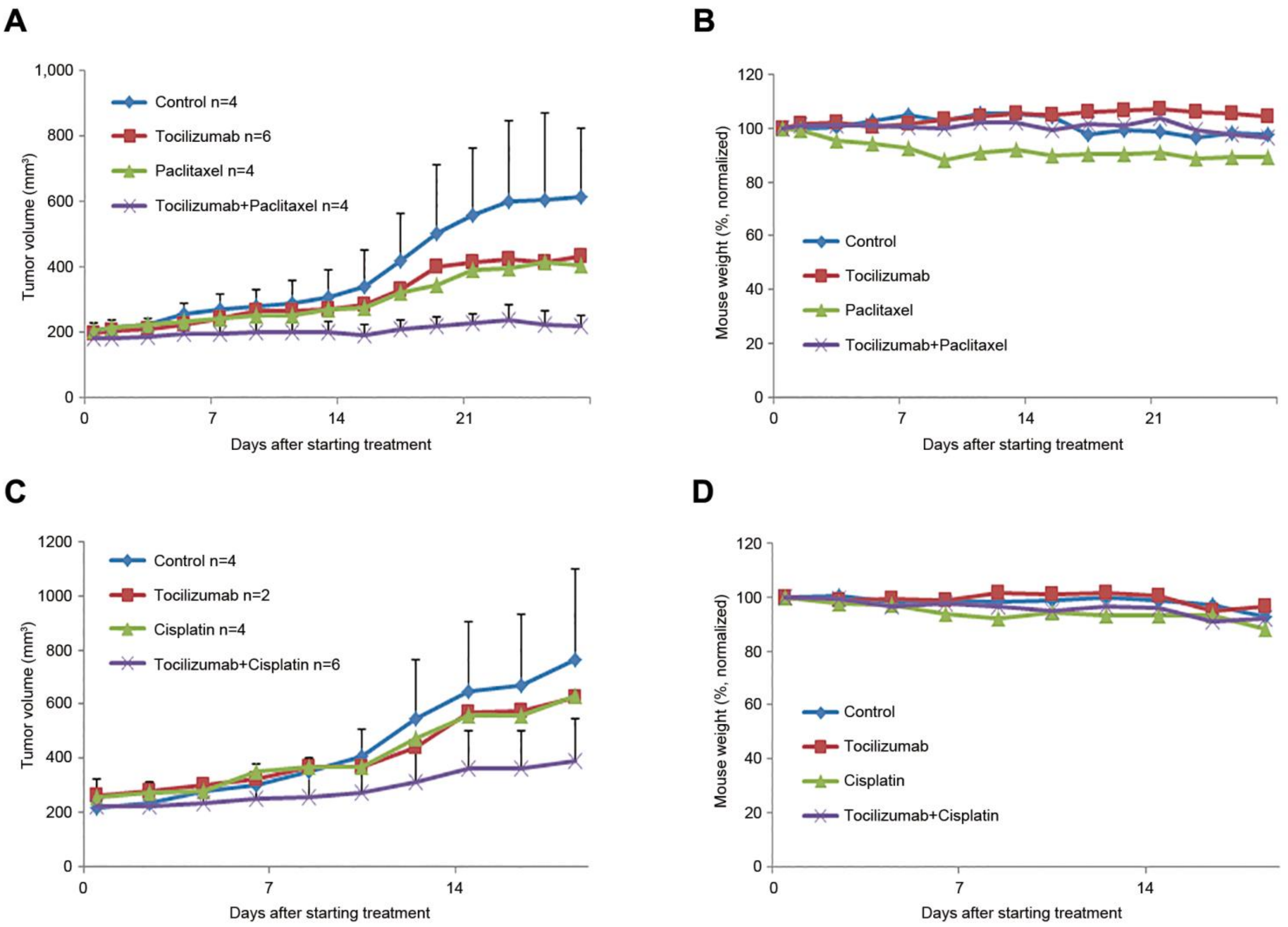

Supplementary Figure S2: Effect of tocilizumab and/or chemotherapy in preclinical models of mucoepidermoid carcinoma. Xenograft tumors were generated in immunodeficient mice upon transplantation of UM-HMC-3B cells. A, graph depicting tumor volume over time of mice treated with tocilizumab and/or paclitaxel. When tumors reached 200 mm^3, mice were randomized into the 4 different treatment regimens as follows: 5 mg/kg IgG control, 5 mg/kg tocilizumab, 20 mg/kg paclitaxel, or 20 mg/kg paclitaxel combined with 5 mg/kg tocilizumab weekly via i.p. injection. B, Graph depicting mouse weight during treatment, from the experiment presented in panel A. Data were normalized against pre-treatment weight. C, graph depicting tumor volume over time of mice treated with tocilizumab and/or cisplatin. When tumors reached 200 mm^3, mice were randomized into the 4 different treatment regimens as follows: 5 mg/kg IgG control, 5 mg/kg tocilizumab, 5 mg/kg cisplatin, or 5 mg/kg cisplatin combined with 5 mg/kg tocilizumab weekly via i.p. injection. D, Graph depicting mouse weight during treatment, from the experiment presented in panel C. Data were normalized against pre-treatment weight.

Treatment	Dose (mg/kg)	Route/Schedule	Tumor Volume Data	
			Mean + SD (mm^3)	TGI (%)
Control	5	Weekly i.p.	592 ± 160	-
Tocilizumab	5	Weekly i.p.	538 ± 373	30.7
Cisplatin	5	Weekly i.p.	526 ± 330	34.3
Paclitaxel	15	Weekly i.p.	453 ± 247	29.9
Tocilizumab + Cisplatin	5, 5	Weekly i.p.	407 ± 129	57.6
Tocilizumab + Paclitaxel	5, 15	Weekly i.p.	331 ± 115	62.1

Supplementary Figure S3: Table depicting tumor growth inhibition (TGI) index in the mucoepidermoid xenograft studies presented in Figures 2 and 3. TGI was determined by the formula: TGI (%) = {1- [(T_F-T_i) ÷ (C_F-C_i)] x 100%} where T_F and T_i are the treated tumor final and initial volumes, and C_F and C_i are the control tumor final and initial volumes.
